# Imaging in the diagnosis of progressive supranuclear palsy

**DOI:** 10.1007/s00415-018-9148-5

**Published:** 2018-12-12

**Authors:** Matthew D. Smith, Stefan Schwartz, Neil P. Robertson

**Affiliations:** 1 Department of Neurology, University Hospital of Wales, Heath Park, Cardiff, CF14 4XN UK; 2 Department of Radiology, University Hospital of Wales, Heath Park, Cardiff, CF14 4XN UK; 3 Department of Neurology, Institute of Psychological Medicine and Clinical Neurosciences, Cardiff University, Cardiff, CF14 4XW UK

## Introduction

Progressive supranuclear palsy (PSP) is a neurodegenerative tauopathy, which in the early phases of the disease can closely mimic idiopathic Parkinson’s disease (PD). More characteristic features including vertical gaze dysfunction may only present later, which frequently leads to a delay in diagnosis. However, early and accurate diagnosis of PSP is of great importance as there are considerable differences in disease course, prognosis and management when compared to other parkinsonian and non-parkinsonian type neurodegenerative diseases. Furthermore, many of these conditions have distinct pathological and prognostic features which will guide treatment strategies. Increasingly sophisticated and informative neuro-imaging techniques are now becoming more widely available and have the considerable advantage of being non-invasive. In this month’s journal club, we examine recent evidence supporting the use of imaging in diagnosing PSP and other neurodegenerative diseases in a series of case–control studies.

## A new MR imaging index differentiation of progressive supranuclear palsy-parkinsonism from Parkinson’s disease

This study recruited consecutive patients from a single movement disorder clinic to investigate the use of morphological MRI features to differentiate PD from PSP and two of its subtypes. It comprised 34 participants with the PSP-P (parkinsonism) phenotype, 46 with PSP-RS (Richardson’s syndrome) and 53 with idiopathic PD and were compared with 53 age/sex-matched controls. Participants were excluded if imaging detected features of normal pressure hydrocephalus, any vascular lesion, or if they had a normal ^123^I-FP-CIT-SPECT scan. Clinical diagnoses were made by a single clinician. Participants underwent Unified Parkinsons Disease Rating Scale (UPDRS) motor examination, mini mental-state examination (MMSE) and Hoehn and Yahr staging. Gaze palsy was quantified according to new PSP clinical diagnostic criteria as “O1” (significant) or “O2” (mid-level).^1^ All patients underwent a 3T MRI scan at study enrolment with automated pons/mid-brain area ratio calculation, comprising the Magnetic resonance Parkinsons ratio (MRPI). Measurements of the third ventricle and frontal horn width were added to form a further “MRPI 2.0” index.

MRPI and MRPI 2.0 significantly differentiated between PD and PSP (*P* < 0.001) being higher in PSP. These indices were also able to differentiate between the PSP-RS and PSP-P subtypes (*P* < 0.001) with indices significantly higher in PSP-RS. Accuracies of 88.5% and 96.6% were achieved for MRPI and MRPI 2.0, respectively, in differentiating PSP-P from PD, and 100% for both indices for PSP-RS versus PD. The accuracy of MRPI and MRPI 2.0 values to classify patients with PSP-P based on the extent of vertical gaze palsy was shown to be 98.5% and 100%, respectively, for “O1” grades and 81.9% and 95.8% for “O2” grades.

*Comment* This paper demonstrates compelling evidence of MRI morphological features to accurately classify patients to PSP and PD groups, as well as specific PSP subgroups. The addition of further morphological features to the automated imaging evaluation (MRPI versus MRPI 2.0) further improved accuracy. The exclusion of individuals with vascular lesions on scans may serve to remove the population from a comparable clinical cohort and could alter the significance of the results. It may also be limited by the assumption that the initial clinical diagnosis to which the MRI scan is correlated was correct and that there was no direct neuropathological confirmation.

Quattrone *et al*. (2018) Parkinsonism Relat Disord. 2018 Sep;54:3–8.

## Improved automatic morphology-based classification of Parkinson’s disease and progressive supranuclear palsy

In this case comparison study, PSP-RS (*n* = 22) and PD (*n* = 76) patients were recruited from a single movement disorder clinic, to determine whether morphological features on MRI combined with clinical features can classify patients correctly via a machine learning algorithm. Data were collected on age, gender, disease duration and participants underwent UPDRS, MMSE and Hoehn and Yahr assessment. PSP participants with features including freezing and levodopa responsiveness were excluded. Participants underwent 3T T1-weighted scans MRI, parcellated to a brain atlas dividing into regions of interest. Surface area and volume for each area was calculated and entered into the complex machine-learning pipeline with the seven clinical features above.

PSP participants were older, with a disease duration of half the PD patients. Twenty-one subcortical regions were assessed in each group (including brainstem, basal ganglia). Volumetric differences in these regions between cohorts alone achieved an accuracy of 84.69% in differentiating PSP-RS versus PD. Adding clinical features increased accuracy to 94.89%. Data from regional surface area alone achieved 85.71% rising to 95.91% with the addition of clinical features. A combination of surface area, volumetric features and a surface area to volume ratio achieved 88.7% accuracy. Adding clinical features to this combination imaging model achieved a 97.95% accuracy. A similar analysis adding 48 cortical regions achieved a similar distribution of accuracies with a 97.95% accuracy achieved using all available features.

*Comment* This study suggests that computer calculated morphological features alone can differentiate PSP-RS from PD cases with considerable accuracy. The addition of quantitative clinical features improves this further. As in the first study, the lack of neuropathological confirmation leaves the standard to which the machine learning protocol is trained and tested dependent on the accuracy of clinical diagnosis. Finally, there are considerable differences in age and disease duration between the cohorts so that it is difficult to rule out an effect of these confounders on the models proposed, particularly in terms of regional brain atrophy.

Talai *et al*. (2018) Clinical Neuroradiology. Sep 14. [E-pub ahead of print]

## ^18^F-AV-1451 positron emission tomography in Alzheimer’s disease and progressive supranuclear palsy

In another case–control study, the authors have investigated the use of the specific tau binding radioligand ^18^F-AV-1451 to differentiate disease in patients with PSP-RS, Alzheimer’s disease (AD) and mild cognitive impairment (MCI). Participants were recruited from the NIMROD (AD, *n* = 9; MCI, *n* = 6), and DeNDRoN (PSP, *n* = 19) projects and compared to controls (*n* = 13). As well as demographic data, patients underwent an Addenbrooke’s cognitive examination (ACE-R), MMSE and PSP rating scale in the PSP cohort specifically. All participants underwent a 3T MRI scan, and a PET-CT scan with injection of the ^18^F-AV-1451 radioligand. Both scans were partitioned into anatomical regions of interest and analysed for ^18^F-AV-1451 uptake in specific regions of interest. Variations in radioligand uptake were then analysed by a machine learning algorithm. A further neuropathological study was undertaken on post-mortem single brains from PSP, AD or healthy subjects. Brains were divided into sections and treated with ^18^F-AV-1451 prior to phosphor screen autoradiography.

Patients were effectively age-matched between groups (*P* = 0.3). Significant regional variations in ^18^F-AV-1451 uptake were found (*P* = 0.00001) based on clinically defined group. AD and MCI participants overall had higher binding, reflecting the greater burden of tau in AD pathology. PSP participants demonstrated increased binding in the midbrain (*P* < 0.04) and pallidum, thalamus, putamen and dentate nucleus of the cerebellum (*P* < 0.02) versus AD/MCI groups and controls. AD/MCI groups demonstrated increased binding in all cortical structures (*P* < 0.04). ^18^F-AV-1451 regional binding was correlated against ACE-R for all groups and PSP rating scale, with no significant difference found suggesting correlation between uptake and disease process severity.

The ability of the machine learning algorithm to classify participants into groups based on scans was then assessed. 94.1% accuracy was achieved in differentiating AD/MCI versus PSP, 85.7% AD/MCI versus control and 90.7% PSP versus control. Finally, the paper looked at the post-mortem brain sections. Neuropathological studies did not show any post-mortem ^18^F-AV-1451 binding specific to PSP.

*Comment* This study is useful in that it demonstrates differences in imaging detectable tau pathology in PSP that is discriminatory between controls and an alternative common neurodegenerative condition. To further elaborate on this work, it would be useful to assess a non tau pathology associated parkinsonian disease cohort like a cohort of PD patients as a clinically relevant comparator group and observe whether regional binding varied. As per other studies, neuropathological evidence is not obtained to confirm diagnostic accuracy for PSP in the clinical component of the study. However, tying the imaging methodology together into the automated machine learning algorithm demonstrated good accuracy in discriminating the different conditions, suggesting that ^18^F-AV-1451 maybe a useful biomarker for detecting PSP.

Passamonti *et al*. (2017) Brain. Mar 1;140(3):781–791

## Conclusion

These studies each demonstrate evidence that imaging modalities such as MRI and tau binding radioligands (F-AV-1451) can accurately differentiate between PD and PSP disease states, and even between PSP subtypes. The majority of this work has involved quantification of imaging features using computerised analysis and, in two cases, machine learning algorithms. All studies lacked a neuropathological diagnostic confirmation for PSP. The result of this is that each study is dependent on the accuracy of the initial assessors recruiting patients to correctly diagnose PSP, which acts as the “ground truth” to which the discriminatory ability of the imaging modalities relies. In common with many case–control studies, there are also a range of potential confounders that need to taken into consideration. Future work to assess the utility of imaging in differentiating PSP from other movement disorders such as PD would likely benefit from engaging large cohorts with patients at a variety of disease stages, carefully matched with control cases and with longitudinal neuropathological confirmation at death.

